# Integrated morphological and transcriptome profiles reveal a highly-developed extrusome system associated to virulence in the notorious fish parasite, *Ichthyophthirius multifiliis*

**DOI:** 10.1080/21505594.2023.2242622

**Published:** 2023-08-07

**Authors:** Hao Yang, Zhe Wang, Jieyin Xiao, Jingbo Hu, Xiao Tu, Zemao Gu

**Affiliations:** a College of Fisheries, Huazhong Agricultural University, Wuhan, China; b Hubei Engineering Technology Research Center for Aquatic Animal Diseases Control and Prevention, Wuhan, China; c National Aquatic Animal Diseases Para-Reference Laboratory (HZAU), Wuhan, China; d Marine College, Shandong University, Weihai, China; e Hubei Hongshan Laboratory, Wuhan, China

**Keywords:** *Ichthyophthirius multifiliis*, morphologenesis, RNA-seq, transcriptome, exosome

## Abstract

*Ichthyophthirius multifiliis* is an obligate parasitic ciliate that causes severe economic damage in aquaculture. The parasite contains numerous extrusive organelles (extrusomes) that assist in its pathogenesis and reproduction. However, the structure of these extrusomes and the molecular profiles involved in exocytosis remain unclear. In the present study, through comparative ultrastructural observations across the life cycle of *I. multifiliis*, we demonstrated that all three of its life stages (theront, trophont, and tomont) exhibited an abundance of extrusomes. In addition, two different types of extrusomes were identified according to their unique structures. Type I extrusomes (mucocysts) are crystalline, oval-shaped, 0.7–1.4 × 0.6–1.1 μm, and distributed as “rosettes” below the trophont membrane. Type II extrusomes, 2.0–3.0 × 0.2–0.3 μm, are rod-shaped with tubular cores and identified as toxicysts, the aggregation of which in the anterior part of the theront and cortex of the trophont revealed their potential roles in *I. multifiliis* invasion. This was confirmed by our transcriptome investigations of the three stages of *I. multifiliis*, which revealed that a set of genes involved in proteolysis and DNA/protein biogenesis was highly expressed in the theront and trophont. Furthermore, to map the molecular mechanisms of extrusome release, we characterized 25 Rab family genes in *I. multifiliis* and determined their expression profiles across the life cycle, reflecting the distribution patterns of the two extrusomes. Collectively, our data revealed that a highly developed extrusome system could play a potential role in the virulence of *I. multifiliis*, which facilitates a better understanding of the parasite’s development.

## Introduction

Extrusomes are a group of membrane-bound secretory organelles that are widely found in ciliated protozoans [1,2]. These vesicular organelles are associated with the cell membrane and have different structures containing materials that are usually expelled or extruded from the cell to engage in different functions [3]. For example, toxicysts are typical offensive extrusomes of ciliates that can extrude toxic material that potentially play a predatory role in prey capture and food uptake [4,5]. Correspondingly, mucocysts can secrete amorphous mucilaginous protective material on the cell surface to form cysts or temporary capsules, contributing to the protection of prey species from predators [6,7]. In addition, extrusomes are present in many parasitic species, and their extrusion assists in the establishment of the parasite within the host by evading the attack of the host immune system [8].

*Ichthyophthirius multifiliis* is an obligate parasitic ciliate that accounts for large-scale mass mortality events in freshwater aquaculture and the ornamental fish industry [9,10]. Its direct life cycle contains parasitic (trophont) and non-parasitic (tomont and theront) phases. At the tomont stage, the parasite must experience multiple divisions to produce infective theronts, thus, to ensure normal reproduction, a mucilaginous cyst wall is secreted by the tomont to anchor itself to substrates [11,12]. Mucocysts are important extrusomes in *I. multifiliis* that enables the formation of the cyst wall and, as suggested by Ewing et al. [13], play a potential role in *I. multifiliis* pathogenesis, as evidenced by the continuous envelopment of the theront during invasion by extrusive materials. However, the existence of other extrusomes types in *I. multifiliis* has not been described up until now. Moreover, the morphological characteristics of extrusomes and molecular profiles related to their release remain unclear.

In the present study, the ultrastructural features of the three life stages of *I. multifiliis* were investigated using scanning and transmission electron microscope observations. Two types of extrusomes were identified in the plasma of *I. multifiliis* and their morphological characteristics were described. Furthermore, a comparative transcription analysis across the life cycle of *I. multifiliis* was conducted to map the molecular information associated with these extrusomes.

## Materials and methods

### Collection of parasite stages

*Ichthyophthirius multifiliis* utilized in this study was originally obtained from a local farm of yellow catfish, *Tachysurus fulvifraco*, in Wuhan City, Hubei Province, China. Healthy goldfish were purchased from a pet store in Shanghai, China. The parasite was maintained in the Fish Disease Laboratory of the College of Fisheries, Huazhong Agricultural University, by serial transmission on goldfish. Mature trophonts were gently scraped from heavily infected goldfish and rinsed several times with dechlorinated water to remove mucus. Clean trophonts were incubated at 25°C to release infective theronts. Theronts were used to challenge goldfish according to the method described by Li et al. [14]. The use of goldfish was approved by the Animal Experimentation Ethics Committee of the Huazhong Agricultural University.

### *Morphological observations on* I. multifiliis

Morphological features were observed with a bright-field differential interference contrast microscope (Olympus BX53F, Japan) under 200 × and 400 × magnifications. Images were captured using a digital camera mounted on a microscope (DP73, Olympus, Japan). Released theronts were collected in 1.5-mL EP tubes and centrifuged at 2,000 rpm to obtain the abundant parasite. Five microlitres of liquid containing the theronts was transferred to the middle of a glass slide, the morphology of the theronts was then observed under 100× magnification, and images were captured.

For the scanning electron microscopy, concentrated trophonts, tomonts, and theronts were fixed overnight in cold (4°C) 2.5% glutaraldehyde. The fixed theronts were pipetted onto a glass slide (6 mm diameter) coated with 1% Poly-Lysine and allowed to settle for 6 h. All specimens were then washed with PBS (0.1 M, without NaCl) 3–5 times and subsequently dehydrated in a graded ethanol series (30%, 50%, 70%, 80%, 90%, 95%, 100%, 100%), followed by two 20-min washes in amyl acetate. Finally, after natural drying, the specimens were sputter-coated with gold and examined using a scanning electron microscope (HITACHI SU-8010, Japan).

For the transmission electron microscopy, the concentrated trophonts, tomonts, and theronts were fixed in cold (4°C) 2.5% glutaraldehyde overnight. All specimens underwent three 15-min washes in 0.1 M PBS (pH 7.4) and were then post-fixed in 1% osmic acid (pH 7.4) for 2 h at room temperature and repeatedly washed with PBS (0.1 M). Thereafter, the specimens were dehydrated in a graded ethanol series (30%, 50%, 70%, 80%, 90%, 95%, 100%, 100%) and infiltrated sequentially with acetone: Epon (2:1 ratio), acetone and Epon (1:1 ratio), and Epon for 12 h at 37°C, respectively. Following embedding in Epon (68°C, 48 h), ultrathin (80–100 nm) sections were cut and then stained with 2% uranium acetate and lead citrate for 15 min at room temperature. Finally, the sections were examined under a transmission electron microscope (FEI Tecnai G^2^ 20 TWIN, USA).

### RNA extraction, library construction and sequencing

Each life stage of *I. multifiliis* had three biological replicates. For each replicate, 30000–50,000 theronts, 300–500 trophonts, and 300–500 tomonts were used. The cells were first suspended in ddH_2_O and then adsorbed on the membrane by suction filtration through 0.22 μm membrane. The cells were transferred to a 2-mL frozen storage tube, and total RNA was extracted using the Trizol reagent kit (Invitrogen, Carlsbad, CA, USA) according to the manufacturer’s protocol. RNA quality was assessed using an Agilent 2100 Bioanalyzer (Agilent Technologies, Palo Alto, CA, USA) and verified by RNase-free agarose gel electrophoresis. After total RNA was extracted, the mRNA was enriched using oligo (dT) beads. The enriched mRNA was fragmented into short fragments using fragmentation buffer and reverse transcribed into cDNA using the NEBNext Ultra RNA Library Prep Kit (NEB #7530, New England Biolabs, Ipswich, MA, USA). The purified double-stranded cDNA fragments were end-repaired, A base was added, and ligated to Illumina sequencing adapters. The ligation reaction was purified using AMPure XP Beads (1.0X). Polymerase chain reaction (PCR) was performed. The resulting cDNA library was sequenced using Illumina Novaseq6000 (Gene Denovo Biotechnology Co., Guangzhou, China). The RNA-seq raw data was submitted to NCBI, with a BioProject ID PRJNA991275.

### Read mapping and data processing

To obtain high-quality clean reads, the reads were further filtered using fastp [15] (version 0.18.0). The parameters were as follows:1) removing reads containing adapters; 2) removing reads containing more than 10% unknown nucleotides(N); and 3) removing low-quality reads containing more than 50% of low-quality (Q-value ≤20) bases. An index of the reference genome was built and paired-end clean reads were mapped to *I. multifilii*s reference genome [9] using HISAT2. 2.4 [16], and other parameters were set as default. The mapped reads of each sample were assembled using StringTie v1.3.1 [17,18] using a reference-based approach. For each transcription region, a fragment per kilobase of transcript per million mapped reads (FPKM) value was calculated to quantify its expression abundance and variations using the RSEM software [19]. Principal component analysis (PCA) was performed using the R package gmodels (http://www.rproject.org/). RNAs differential expression analysis was performed using DESeq2 [20] software between two different groups and edgeR [21] between the two samples. Genes/transcripts with a false discovery rate (FDR) below 0.05 and absolute fold change ≥ 2 were considered differentially expressed genes/transcripts. The differently expressed genes (DEGs) were mapped to GO and pathway terms. Gene numbers were calculated for every term, and significantly enriched terms in DEGs compared to the genome background were defined using a hypergeometric test. Gene set enrichment analysis was performed using GSEA and MSigDB software [22] to identify whether a set of genes in specific GO terms/KEGG pathway terms showed significant differences between the two groups. Rab proteins constitute the largest family of small GTPases, which are coupled with guanosine-5’-triphosphate (GTP) binding and hydrolysis processes at different stages of vesicle transport [23,24]. For Rab-related gene (gene annotated to Rab proteins) family characterization, the conserved domains were demonstrated in NCBI, and the motif patterns were analysed using MEME version 5,5,2 [25], of which the maximum number was set to 10. To study the phylogenetic relationships of the Rab genes in *I. multifiliis*, multiple sequence alignments of amino acid sequences were performed using the ClustalW program with default parameters. An un-rooted Maximum Likelihood (ML) tree was constructed with 1000 bootstrap replications using MEGA 7.0 [26] software, based on full-length protein sequence alignment.

## Results

### Morphological features in theront

**Figure 1.**
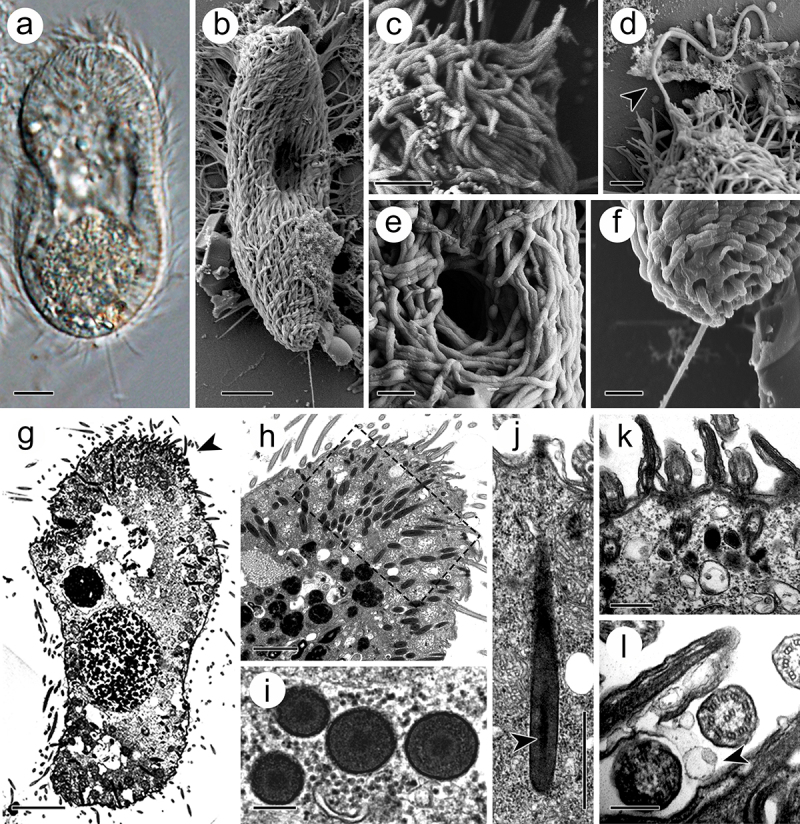
Theront of *I. multifiliis*. (a, b) Holistic view of the theront. (c, d) Magnified perforatorium at the apex. Arrowhead indicates the extrusive contents. (e) Cytostome. (f) Caudal cilium at the terminal end. (g) Longitudinal section views of theront. Arrowhead indicates the anterior part. (h) Cytoplasm of the anterior part of theront. (i) Cross section of toxicyst. (j) Longitudinal view of toxicyst showing the tube-like core (arrowhead) located within the capsule and a fibro-granular layer at the posterior end. (k) Cortex of theront. (l) Extrusion of vacuole from cilia membrane. Arrowhead indicates the ejected vacuole. Scale bars 5 μm (a, b, g), 2 μm (c, d, h), 1 μm (e, f, j), 500 nm (k), 200 nm (i, l).

The theront, 50–70 μm in length and 30–50 μm in width, was initially spindle shaped and eventually transitioned into a sole shape after a period of swimming life (Figure 1a,b). At its anterior end, the somatic cilia converged into bundles, which covered the perforatorium (Figure 1c). The latter could discharge rod-like materials from its apices (Figure 1d). The circular cytostome, 2–5 μm in diameter, was located in the anterior part of the body, 10–20 μm from the anterior end (Figure 1e). At the posterior end, the theront developed a rigid caudal cilium of 10–15 μm in length, which is double to triple the length of the somatic cilia (approximately 5 μm) (Figure 1f). In the cytoplasm, numerous electron-dense extrusomes, which are spindle-shaped and measured 2–3 μm in length and 0.2–0.3 μm in width, were distributed and oriented towards the perforatorium (Figure 1g,h). Morphologically, the extrusome consists of an electron-dense capsule wall, measuring 10–15 nm in width, and a tubular filament inside the intracapsular matrix (Figure 1h,j). At cross sections, the filament, 0.10–0.14 μm in diameter, displayed a less electron-dense periphery and an irregularly shaped internal electron-dense matrix (Figure 1i). At the basal end of the extrusomes, the capsule wall contained a fibro-granular layer (FGL) with a homogeneous appearance (Figure 1j). In addition, abundant membrane-coated vesicles were distributed throughout the cytoplasm and extruded through the three-unit cell membrane (Figure 1k,i).

### Morphological features in trophont

**Figure 2.**
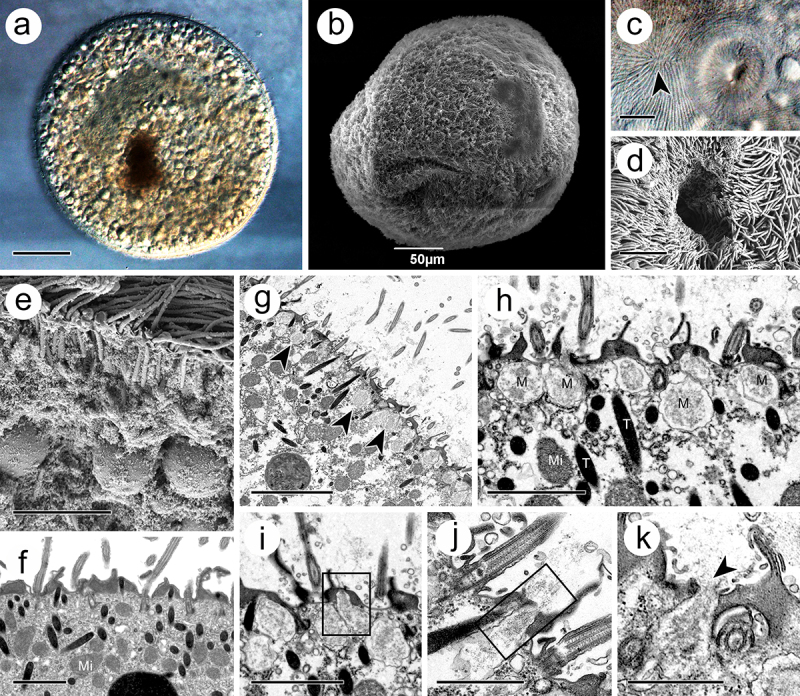
Trophont of *I. multifiliis*. (a, b) Holistic view of trophont. (c, d) Cytostome with nearly apex of trophont (arrowhead) where kineties rationally extended. (e, f) Cortex periphery of trophont. (g) Rosettes of mucocyst (arrowheads) contains amorphous materials laid down the cell membrane. (h) Magnification of mucocyst. (i – k) Mucocysts at different extrusive stage. M, mucocyst; Mi, mitochondrion, T, toxicyst. Scale bars 50 μm (a, b), 20 μm (I), 10 μm (c), 5 μm (d, e, g) 2 μm (f, h, i), 1 μm (j, k).

The trophont was spindle- to round-shaped, with a length of 340–470 μm and a width of 290–440 μm (Figure 2a). Holistically, the surface of the trophont exhibited an abundance of densely distributed cilia (Figure 2b). The cytostome, 15–20 μm in width and 15–25 μm in depth, was found near the anterior end (Figure 2c,d). At the cytoplasm periphery, the trophont was filled with food vacuoles, which were characterized by undigested host cells in matrices (Figure 2e). Numerous electron-dense extrusomes, with the same organization as those in the theront, were distributed in a disorderly manner and with various orientations (Figure 2f–h). Another type of extrusome, characterized by a spherical shape, 0.7–1.4 × 0.6–1.1 μm, and arrayed as “rosettes” around ciliary insertions, was observed suspended beneath the plasma membrane (Figure 2i). During the release process of the extrusome, the membrane of these organelles would fuse with the cell membrane, thus expelling their internal substances (Figure 2j,k).

### Morphological features in tomont

**Figure 3.**
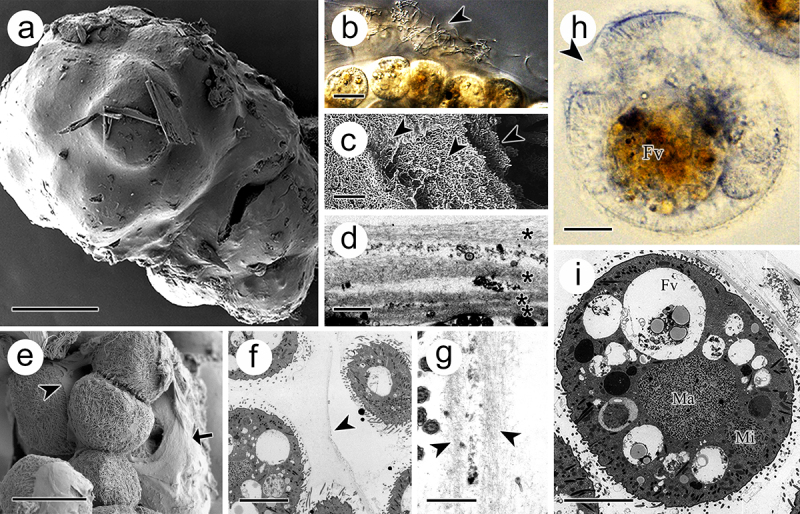
Tomont of *I. multifiliis*. (a) Holistic view of the tomont enveloped by cyst wall. (b) the cyst wall trapped with bacteria and debris (arrowhead). (c, d) Multi-layers of the cyst wall. Arrowheads and “*” indicated the separated layers. (e) Dividing offspring. Arrow indicates the cyst wall and arrowhead indicates the inner membrane. (f and g) Inner membrane (arrowheads) of tomont. (h, i) Holistic view of the offspring in tomont. arrowhead showed the cytostome. FV, food vacuoles; Ma, macronucleus; Mi, micronucleus. Scale bars 50 μm (a, e), 20 μm (b), 10 μm (f, h, i), 1 μm (d, g), 500 nm (c).

The tomont was surrounded by a sticky cyst wall (Figure 3a,b). From the section view, the cyst wall consisted of multiple layers, which were separated by filaments or bacteria (Figure 3c,d). The first two layers were approximately 1 μm, while the following layers were no more than 0.5 μm (Figure 3e). In addition, each offspring was covered with a thin adhesive membrane (Figure 3f). The inner membrane separating the dividing offspring comprised two unit layers (Figure 3g,h). The offspring inside the tomont were ball-shaped and measured 30–50 μm prior to release (Figure 3i). The macronucleus (approximately 15 μm in diameter) was in the middle of the cell, whereas the micronucleus (approximately 10 μm in diameter) was observed adjacent to the cell membrane (Figure 3i).

### DEGs between adjacent life stages

**Figure 4.**
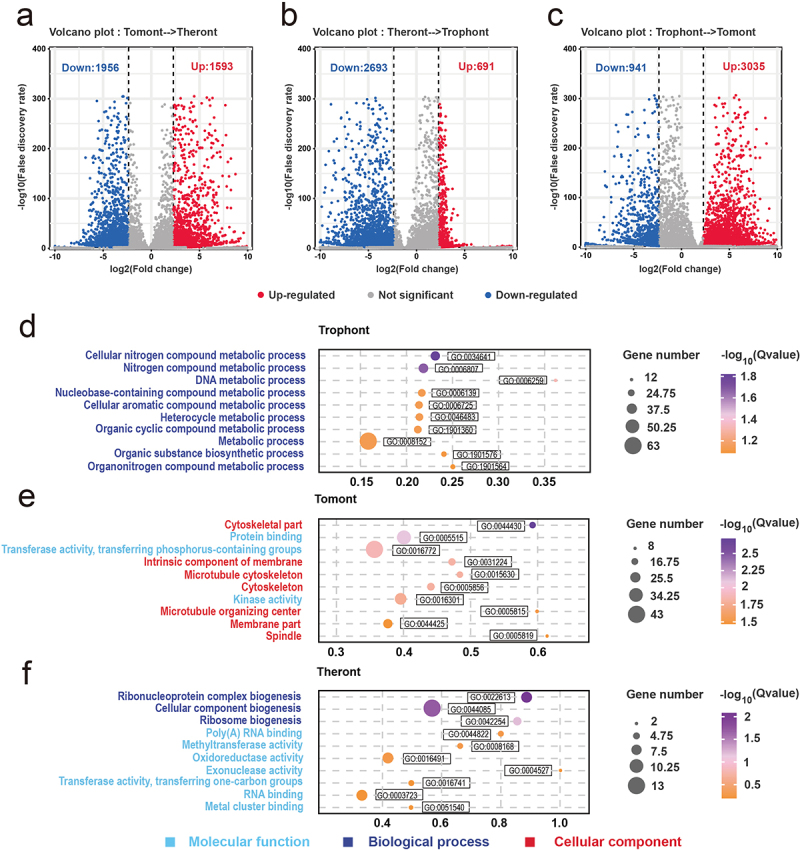
Differential gene expression among the stage. (a – c) Volcano plots showing differentially expressed genes (DEGs) in theront compared to tomont, in trophont compared to theront, and in tomont compared to trophont, respectively. (d – f) GO enrichment analysis based on the up-regulated DEGs in trophont, tomont, and theront, respectively. Gene ratio is the percentage of total DEGs in the given GO term.

Concerning transcript analysis, the PCA plot showed the top two principal components that explained much of the variance between samples in the dataset: 82.8% and 17.1% for PC1 and PC2, respectively. Further, replicates from the theront, trophont, and tomont were clustered, respectively, away from the other stages (Fig. S1a, b). In total, 9,093 genes were identified, with 8,659, 8,477, and 8,714 genes found in the theront, trophont, and tomont, respectively (Fig. S1c). To view the transcriptional changes during each transition between stages, pairwise differential gene expression analysis was performed on the adjacent life stages. In total, 3,549 DEGs were associated with development from tomont to theront, of which 1,593 and 1,956 were upregulated and downregulated, respectively (Figure 4a). Further, 3,384 DEGs were identified between the theront and trophont, of which 691 were upregulated and 2,693 were downregulated (Figure 4b). Moreover, 3,976 DEGs were identified between the trophont and tomont, of which 3,035 and 941 were upregulated and downregulated, respectively (Figure 4c).

From the tomont to the theront, the FDR of the top 10 GO terms were mainly annotated to molecular functions and biological processes, the first three of which were ribonucleoprotein complex biogenesis (GO:0022613), cellular component biogenesis (GO:0044085), and ribosome biogenesis (GO:0042254), respectively. Meanwhile, other functions were mainly presented as enzyme activity, such as methyltransferase (GO:0008168), oxidoreductase (GO:0016491), and exonuclease activity (GO:0004527) (Figure 4d). GO analyses showed that most upregulated genes in the trophont stage were annotated to metabolic processes, including nitrogen compound processes (GO:0034641 and GO:0006807) and organic compound processes (GO:1901360, GO:1901576, and GO:1901564) (Figure 4e). However, the annotation of the upregulated DEGs in the tomont stage differed from that in the other two stages, whereas seven of the top 10 GO groups were annotated as cellular components (Figure 4f). Among them, three terms (GO:0005856, GO:0015630, and GO:0044430) were related to the microtubular cytoskeleton, indicating its importance in the formation of daughter cells in the tomont. In addition, two terms (GO:0031224 and GO:0044425) were associated with membrane organization (Figure 4f).

**Figure 5.**
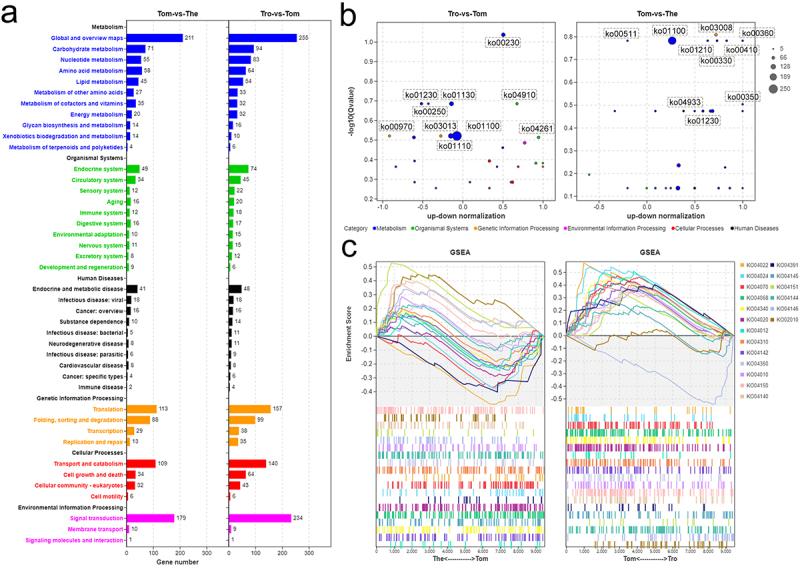
KEGG analysis on the DEGs of *I. multifiliis*. (a) an overview of annotated pathways in trophont-tomont, and tomont-theront. (b) the number of up-regulated and down-regulated genes in top 30 -log_10_(Qvalue) pathways. (c) GSEA analysis genes related to pathways of transport and catabolism, signal transduction, and membrane transport.

To illustrate better the functions of the DEGs in relation to the theront and tomont, KEGG analyses of these DEGs was performed. The general annotations of the trophont – tomont and tomont – theront DEGs were consistent, among which the top three pathways were associated with metabolism, signal transduction, and translation (Figure 5a). Further, to determine whether the associated pathways were activated or inhibited, we compared the number of upregulated and downregulated genes among the top 30 log_10_(Q value) pathways. We found that more genes related to the metabolism pathway were downregulated in the tomont stage, whereas those participating in the organism system or cellular processes were mostly upregulated (Figure 5b). Comparatively, metabolism-associated DEGs were mostly upregulated in the theront stage, which may contribute to a subsequently fast swimming life (Figure 5b). GSEA based on cellular process-related pathways was performed to map the extrusion process in *I. multifiliis*, and we identified 19 pathways associated with transport and catabolism, signal transduction, and membrane transport that showed various degrees of expansion in the theront and tomont stages (Figure 5c).

### Expression profiles of Rab family related genes

**Figure 6.**
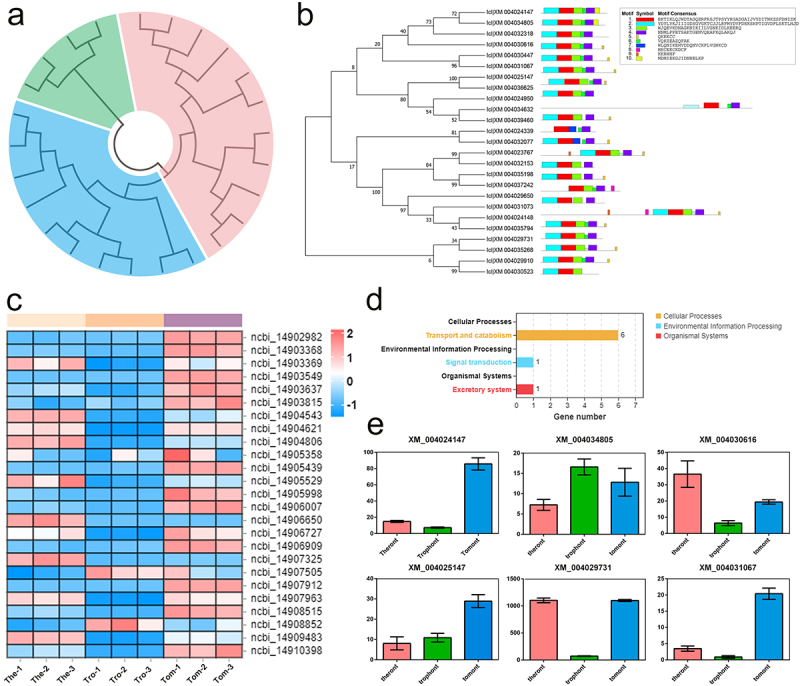
Characterization and expression patterns of Rabs gene family in *I. multifiliis*. (a) an un-rooted ML phylogenetic tree based on amino acid sequences of *I. multifiliis* Rabs. (b) the motif composition of *I. multifiliis* Rabs. The motifs are displayed in different coloured boxes. (c) Expression profiles of Rab-related genes at different stages of *I. multifiliis*. (d) KEGG analysis of the 25 Rab-related genes in *I. multifiliis*. (e) Expression profiles of six selected Rab-related genes at different stages of *I. multifiliis*.

In total, 51 putative genes annotated to Rab were differentially expressed in the transcriptome profiles, but by analysing the conserved domains of these sequences, 25 Rabs were identified (Table 1). To identify the phylogenetic relationship among the 25 Rabs, amino acid sequences were aligned and an un-rooted ML tree was constructed. As shown in Figure 6a and b, *I. multifiliis* Rabs were classified into three groups. A schematic representing the structure of all Rabs was constructed from the MEME Suite’s motif analysis results, which showed that Rabs within the same group usually share a similar motif composition (Figure 6b). Rab-specific motifs including IGVDF, RFRSIT, YYRGA, and LVYDIT, were detected. To illustrate the expression patterns of these Rabs in *I. multifiliis*, a heat map based on their gene expressed fpkm values was created, which showed that 9, 2, and 14 were highly expressed in the theront, trophont, and tomont stages, respectively (Figure 6c). KEGG analyses of these Rab-related genes suggested that six of the 25 were annotated to pathways for transport and catabolism (Figure 6d). The specific expression profiles of these six genes are shown in Figure 6e, and most of them (5/6) were highly expressed in the theront or tomont stage.

**Table 1.** Twenty-five Rab family genes expressed profiles in *Ichthyophthirius multifiliis.*

Gene ID	Average fpkm			NCBI accession number	Gene description
	Theront	Trophont	Tomont		
ncbi_14902982	52.32	66.92	362.59	XM_004023767.1	Rab-family small GTPase
ncbi_14903368	14.90	7.28	85.72	XM_004024147.1	Rab-family small GTPase Rab11A
ncbi_14903369	96.54	20.14	74.77	XM_004024148.1	Rab-family small GTPase
ncbi_14903549	6.14	8.05	243.90	XM_004024339.1	Rab-family small GTPase RabX33
ncbi_14903637	12.66	4.89	27.77	XM_004024950.1	small guanosine triphosphatase family Ras-related in brain (Rab) family protein
ncbi_14903815	8.10	10.92	28.90	XM_004025147.1	Rab-family small GTPase
ncbi_14904543	16.67	0.67	7.38	XM_004029650.1	rab-GTPase-TBC domain protein
ncbi_14904621	1102.96	72.65	1099.39	XM_004029731.1	rab_A30
ncbi_14904806	446.11	11.31	186.33	XM_004029910.1	Rab-family small GTPase
ncbi_14905358	1.37	1.35	1.76	XM_004030447.1	small guanosine triphosphatase family Ras-related in brain (Rab) family protein
ncbi_14905439	23.72	22.60	108.58	XM_004030523.1	Rab-family small GTPase
ncbi_14905529	36.57	6.38	19.41	XM_004030616.1	Rab-family small GTPase
ncbi_14905998	3.77	1.94	26.28	XM_004031073.1	Rab-family small GTPase
ncbi_14906007	3.47	0.90	20.38	XM_004031067.1	Rab-family small GTPase Rab8E
ncbi_14906650	80.85	4.60	6.15	XM_004032077.1	Rab-family small GTPase
ncbi_14906727	20.99	6.54	24.81	XM_004032153.1	Rab-family small GTPase
ncbi_14906909	2.61	3.24	36.63	XM_004032318.1	small guanosine triphosphatase family Ras-related in brain (Rab) family protein
ncbi_14907325	168.37	6.63	22.98	XM_004034632.1	Rab-family small GTPase Rab2E
ncbi_14907505	7.25	16.60	12.83	XM_004034805.1	small GTPase family Rab protein
ncbi_14907912	32.43	20.21	1257.32	XM_004035198.1	Rab-family small GTPase
ncbi_14907963	26.89	1.54	24.95	XM_004035268.1	small guanosine triphosphatase family Ras-related in brain (Rab) family protein
ncbi_14908515	42.12	11.66	137.01	XM_004035794.1	Rab-family small GTPase
ncbi_14908852	33.06	53.56	39.38	XM_004036625.1	Rab-family small GTPase
ncbi_14909483	78.68	3.44	47.90	XM_004037242.1	Rab-family small GTPase Rab6C
ncbi_14910398	67.50	5.27	171.88	XM_004039460.1	Ras-related protein Rab-2

## Discussion

As a highly virulent pathogen in aquaculture, *I. multifiliis* is known for its serious impact on freshwater fish [10]. Thus, in the present study, we performed an integrated investigation of the life cycle of *I. multifiliis*, whereby morphological characteristics and gene expression profiles were described. Given that extrusome exocytosis in *I. multifiliis* is important yet rarely studied, we focused primarily on the parasite’s internal organization of extrusomes, the potential functions and extrusive mechanisms of which were also discussed.

The theront is the only infective stage of *I. multifiliis*, which following initial contact with the fish epithelium, may enter the host by burrowing between epithelial cells [13]. Accordingly, the perforatorium is a feature of *I. multifiliis* important to this process, but limited information is available about its morphology [11]. Herein, we first identified that the perforatorium retained its secretory ability, as it was able to discharge rod-like materials, but these materials’ origin and functions raised questions. To address this, we focused on the theront cortex, where we notably found numerous extrusomes with high electron-density arranged in the anterior part of the theront and orientated to the perforatorium. Considering that the extrusomes harboured tubular contents similar to those secreted by the perforatorium, we suggested these organelles are involved in invasion of the theront. This is consistent with the previous view that materials from these extrusomes might aid in adhesion to the outer surface of the fish host [12]. So to illustrate better the roles of these extrusomes, their morphological characteristics were further determined in the present study.

Extrusomes are known for their ejection capacity, which can extrude the matrix when stimulated [1]. Herein, elongated electron-dense, tube-like contents and extrusive organelles were distributed throughout the cytoplasm of the theront and trophont. Chapman and Kern [27] termed these extrusomes “mucocysts,” which have with a specialized structure differing from that of other ciliates. This is supported by Ewing et al. [13], who noted that two types of mucocysts exist in *I. multifiliis*. We compared the characteristics of the extrusome with those of *Tetrahymena*, and found significant differences in their locations (cytoplasm vs. basal cilium), electronic densities (dense vs. transparent), and morphologies (with core vs. without core) [28–30]. Instead, the extrusome most resembled toxicyst, as both have a rod-like shape, both are composed of an external capsule and an internal tube-like bore, and both bear an FGL at the apex [1]. Thus, we propose herein that these extrusomes are toxicysts. As such, to the best of our knowledge, there are no reports on extrusomes other than mucocysts in *I. multifiliis*.

The identification of toxicysts in *I. multifiliis* could facilitate a better understanding of parasite – host interactions during the invasion and feeding processes, as toxicysts, which are standard offensive extrusomes of predator species, are known for their poisonous content [1]. Further, our data suggest that the theront perforatorium may also be a channel for the discharge of these extrusomes, which may enable the hydrolyzation of host cells, and which can explain why focal necrosis is accompanied by invasion of the theront [13]. This supports the speculation of Matthews and Matthews [31] that these organelles could be a source of the hyaluronidase detected in theront water. Toxicysts are also present in the trophont, widely distributed throughout the cortex periphery. We further observed that toxicysts can be extruded through the trophont membrane via exocytosis to a certain extent, which can explain why the trophont can induce necrosis of the surrounding tissue cells [32]. Altogether, these observations demonstrate that toxicysts appear to assist in feeding the host cells in *I. multifiliis* [4].

Numerous studies have demonstrated that mucocyst, another type of extrusome in *I. multifiliis*, is involved in the formation of the tomont’s cyst wall [13]. Ewing et al. [33] suggested that this wall comprises a homogeneous inner layer and an electron-transparent outer layer. Herein, we found this structure to be composed not of two but of more than four layers, a feature that effectively protects the dividing daughter cells from fragments or bacteria in water [33]. Meanwhile, highly discriminative vision focused on the surface of the tomont was provided, which indicated that the cyst wall layers were loose fibrous networks. We speculate that this characteristic guaranteed the exchange of materials from water to tomonts, such as oxygen [9]. In addition, we noticed that daughter cells could also secrete the mucus wall, separating them from each other and resulting in numerous dividing groups, a phenomenon is frequently observed after the tomont enters the 32-cell phase. We propose that these complex inner membranes act as protection mechanisms in reproduction, ensuring that the dividing groups are not influenced by each other [34]. This explains why the cyst wall can be slightly damaged without interfering with division and the production of infective theronts [11,33]. Yet, it is still unknown whether the mucocysts are involved in the invasion of *I. multifiliis* as we did not find mucocysts in the theront. However, the abundance of mucocysts in the trophont may indicate their contribution to evasion by the host’s immune system. Further studies focusing on the isolation of these extrusomes are required to elucidate their functions.

Transcript profiles also provide insights into the extrusome exocytosis mechanism of *I. multifiliis*, as we noticed that many genes associated with enzyme function were highly expressed in the theront stage, confirming detection of the lytic enzyme and hyaluronidase activity in water at the molecular level [11]. This further supports our speculation on the structural basis of theront invasion. We hypothesized that the theront releases these enzymes to induce necrosis of fish epithelial cells. Similar mechanisms have been reported in other parasites, including *Taxoplasma* [35], *Plasmodium* [36], and *Trypanosoma* [37]. In addition, we found 14 proteins in the leishmanolysin family specifically expressed in the theront and trophont stages (Table S1). As the major surface antigen of *Leishmania*, leishmanolysin, a critical virulence factor in various *Leishmania* species, was found to bind to the cell membrane through a glycosylphosphatidylinositol (GPI) anchor [38–40]. Similarly, many GPI-anchored proteins are present on the surface of *I. multifiliis*, indicating that leishmanolysin might also be a virulence factor of *I. multifiliis* [41]. As an important metalloprotease, leishmanolysin can manipulate the host’s immune system to allow the parasite to establish, survive, and propagate [42]. Thus, given that *I. multifiliis* also faces a strong immune response from the host during the invasion process, we propose that leishmanolysins may be involved in *I. multifiliis* establishment [8,41]. Interestingly, a recent study showed that leishmanolysin can be released through the exosomes of *Leishmania*, so in view of the highly-developed extrusome systems in *I. multifiliis*, we speculated that toxicysts or mucocysts were involved in leishmanolysins discharge [40,43].

Rab proteins are commonly associated with the cytoplasmic faces of different organelles and the transport vesicles that connect them, suggesting the possible role of Rabs in extrusome extrusion from *I. multifiliis* [44]. In this study, we identified 25 Rabs in *I. multifiliis*, which is more than that identified in other single-cell organisms, including *Trypanosoma bruce* (16) and *Plasmodium falciparum* (11), but almost half of that identified in *Tetrahymena thermophila* (56) [45]. The relatively large number of Rabs in *I. multifiliis* is consistent with the fact that *I. multifiliis* developed an abundance of extrusomes. In addition, we found that 92% (23/25) of Rab-related genes were highly expressed in the theront and tomont, supporting our morphological insights that extrusomes participate in *I. multifiliis* invasion and reproduction. Given that the theront and tomont possess distinct extrusome systems, we hypothesized that the highly expressed Rab-related genes in different stages were related to different extrusive mechanisms. However, further studies on extrusomes transport in *I. multifiliis* at the protein level are needed to verify this hypothesis.

## Conclusion

In conclusion, our data provide a comprehensive description on the morphology and transcriptome profiles of *I. multifiliis* across its life cycle. A highly developed extrusome system was identified and a novel extrusome toxicyst was proposed. Combined with the molecular profiles, we suggest that these extrusomes play a potential role in the virulence of *I. multifiliis*. These data facilitate a better understanding of the development and pathogenesis of this parasite.

## Supplementary Material

Supplemental Material

## Data Availability

Data will be made available from the corresponding author by request.
