# Supplementary material for: Integrated morphological and transcriptome profiles reveal a highly-developed extrusome system associated to virulence in the notorious fish parasite, Ichthyophthirius multifiliis
Source: Virulence. 2023 Aug 7;14(1):2242622. doi: 10.1080/21505594.2023.2242622 (PMC10411306; doi:10.1080/21505594.2023.2242622)
Supplement: Supplemental Material [file KVIR_A_2242622_SM6355.zip › Table S1 (4).docx]

**Table 1. Specially expressed genes (Top 10 high expressed) in theront and tomont**

| **Gene ID** | **Average fpkm** | **Average count** | **Symbol** | **Gene description** |
| --- | --- | --- | --- | --- |
| Tomont |  |  |  |  |
| ncbi_14907586 | 50.49 | 94.33 | XM_004034884.1 | myb-like DNA-binding domain protein, partial |
| ncbi_14905076 | 10.52 | 22.67 | XM_004030153.1 | MORN motif protein |
| ncbi_14906164 | 9.71 | 20.33 | XM_004031241.1 | leishmanolysin family protein |
| ncbi_14910016 | 3.64 | 14.33 | XM_004039005.1 | tubulin-tyrosine ligase family protein |
| ncbi_14906841 | 1.31 | 13.00 | XM_004032269.1 | hydrocephalus-inducing protein |
| ncbi_14907666 | 6.58 | 9.67 | XM_004034944.1 | kelch motif protein |
| ncbi_14909749 | 4.73 | 8.33 | XM_004037496.1 | n-terminal domain protein |
| ncbi_14910169 | 7.64 | 8.00 | XM_004039237.1 | MFS transporter |
| ncbi_14907758 | 2.77 | 7.33 | XM_004035051.1 | PHR domain protein |
| ncbi_14907078 | 4.86 | 7.00 | XM_004032485.1 | inorganic polyphosphate/ATP-NAD kinase |
| Theront |  |  |  |  |
| ncbi_14906845 | 59.65 | 128.67 | XM_004032257.1 | alpha/beta fold hydrolase |
| ncbi_14910953 | 12.79 | 23.67 | XM_004040015.1 | hypothetical protein IMG5_002320 |
| ncbi_14905379 | 6.97 | 15.76 | XM_004030467.1 | zinc carboxypeptidase family protein |
| ncbi_14905551 | 10.48 | 12.00 | XM_004030637.1 | myb-like DNA-binding domain protein, partial |
| ncbi_14902919 | 6.81 | 11.00 | XM_004023706.1 | leishmanolysin family protein |
| ncbi_14906581 | 1.26 | 10.67 | XM_004032008.1 | leishmanolysin family protein |
| ncbi_14904965 | 5.76 | 8.33 | XM_004030066.1 | lipase family protein |
| ncbi_14903765 | 1.76 | 5.67 | XM_004025095.1 | immobilization antigen isoform |
| ncbi_14906708 | 4.61 | 5.33 | XM_004032135.1 | divergent AAA domain protein |
| ncbi_14908181 | 2.09 | 5.33 | XM_004035466.1 | hypothetical protein IMG5_098640 |
