# Supplementary figures and images for: Integrated morphological and transcriptome profiles reveal a highly-developed extrusome system associated to virulence in the notorious fish parasite, Ichthyophthirius multifiliis
Source: Virulence. 2023 Aug 7;14(1):2242622. doi: 10.1080/21505594.2023.2242622 (PMC10411306; doi:10.1080/21505594.2023.2242622)

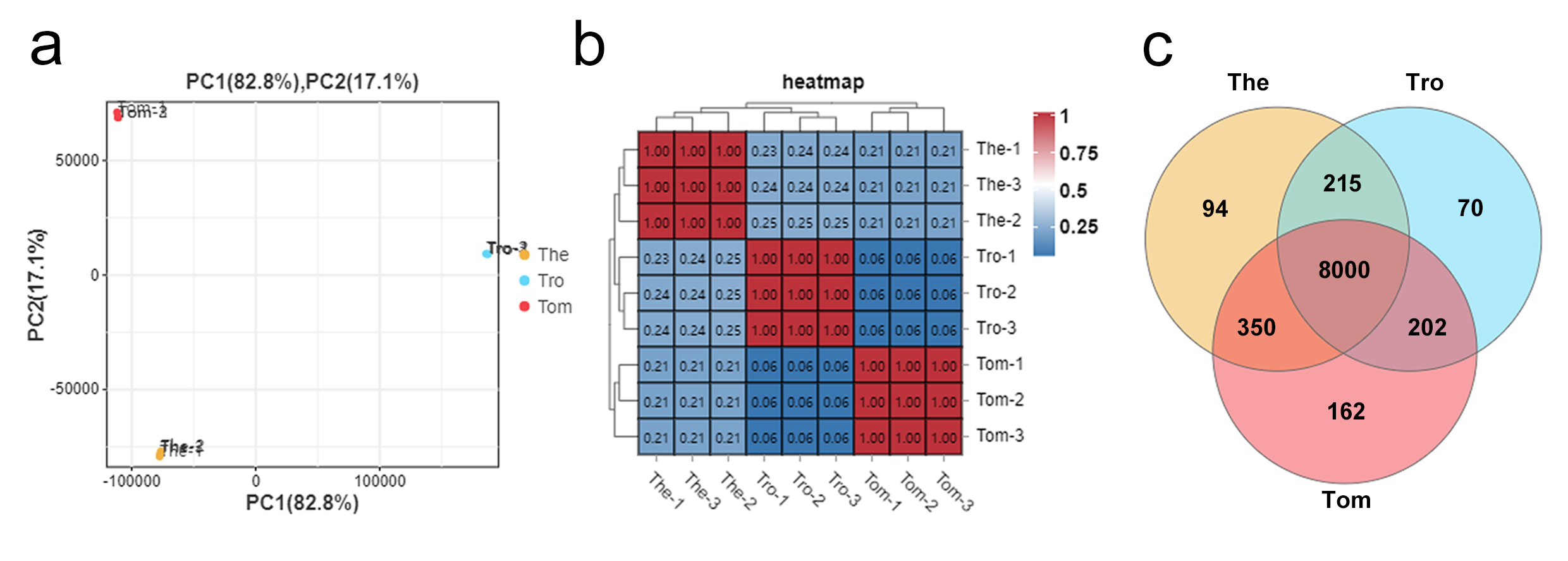

Supplement: Supplemental Material [file KVIR_A_2242622_SM6355.zip › Fig. S1.jpg]
